# Fetal Human Cytomegalovirus Transmission Correlates with Delayed Maternal Antibodies to gH/gL/pUL128-130-131 Complex during Primary Infection

**DOI:** 10.1371/journal.pone.0059863

**Published:** 2013-03-29

**Authors:** Daniele Lilleri, Anna Kabanova, Maria Grazia Revello, Elena Percivalle, Antonella Sarasini, Emilia Genini, Federica Sallusto, Antonio Lanzavecchia, Davide Corti, Giuseppe Gerna

**Affiliations:** 1 Laboratori Sperimentali di Ricerca, Area Trapiantologica, Fondazione Istituto Ricovero e Cura a Carattere Scientifico Policlinico San Matteo, Pavia, Italy; 2 Institute for Research in Biomedicine, Bellinzona, Switzerland; 3 SC Ostetricia e Ginecologia, Fondazione Istituto Ricovero e Cura a Carattere Scientifico Policlinico San Matteo, Pavia, Italy; 4 SS Virologia Molecolare, SC Virologia e Microbiologia, Fondazione Istituto Ricovero e Cura a Carattere Scientifico Policlinico San Matteo, Pavia, Italy; The University of Hong Kong, Hong Kong

## Abstract

Primary human cytomegalovirus (HCMV) infections during pregnancy are associated with a high risk of virus transmission to the fetus. To identify correlates of intrauterine HCMV transmission, serial serum samples from HCMV transmitter and non-transmitter pregnant women with primary HCMV infection were analyzed for the presence of neutralizing antibodies against different glycoproteins and glycoprotein complexes, which are known to mediate entry into distinct types of host cells. Neutralizing activity was detected in the sera early after primary infection; absorption with a soluble pentameric complex formed by gH/gL/pUL128-131, but not with gH/gL dimer or with gB, abolished the capacity of sera to neutralize infection of epithelial cells. Importantly, an early, high antibody response to pentamer antigenic sites was associated with a significantly reduced risk of HCMV transmission to the fetus. This association is consistent with the high *in vitro* inhibition of HCMV infection of epithelial/endothelial cells as well as cell-to-cell spreading and virus transfer to leukocytes by anti-pentamer antibodies. Taken together, these findings indicate that the HCMV pentamer complex is a major target of the antibody-mediated maternal immunity.

## Introduction

Human cytomegalovirus (HCMV) is the most common cause of congenital infection, leading to sensorineural hearing loss and neurodevelopmental delay [1]. The birth prevalence of congenital HCMV infection is estimated to be 0.6–0.7%, with a 11–13% symptomatic newborns rate at birth [2], [3]. The rate of transmission *in utero* is much higher (32.3% *vs* 1.4%) for primary *versus* non-primary infections [2]. In a recent study on 735 pregnancies complicated by primary HCMV infection during a 20-year period it was found that the overall rate of vertical transmission was 37.1%, and ranged from 5.6% for pre-conceptional progressively up to 64.1% for third trimester infections [4]. In addition, primary infection in early gestation carries the highest risk of symptomatic infection in the infected fetuses and newborns [5], [6].

The mechanisms of protection from vertical transmission remain to be elucidated, although the role of T cells in controlling HCMV infection is well established [7]–[10]. In addition to T cells, antibodies may play a role in controlling vertical transmission. In this respect, Nigro et al. reported that HCMV-specific hyperimmunoglobulin preparations appeared to be effective in both prevention of fetal infection and treatment of fetuses infected *in utero* [11]. In addition, it was suggested that maternal antibodies may enhance or prevent fetal infection according to their low or high neutralizing activity [12].

The major targets of the neutralizing antibody response to HCMV are the glycoproteins gB, gM/gN and gH/gL/gO, which mediate entry into host cells [13]. However, we recently found that human monoclonal antibodies neutralize HCMV infection by targeting a protein complex that includes gene products of the HCMV UL128-131 locus [14], which is known to be indispensable for virus growth in endothelial cells and virus transfer to leukocytes [15]. These antibodies were found to be a thousand-fold more potent in neutralizing virus infection of epithelial/endothelial cells as compared to antibodies directed against gB. The UL128-131 gene products are assembled with gH and gL to form a 5-protein (pentamer) complex (gH/gL/pUL128-130-131), which is an alternative to the classical gH/gL/gO complex [16]. Accordingly, the pentamer complex confers to HCMV the ability to infect endothelial and epithelial cells as well as myeloid cells [16]–[19]. Furthermore, gB and gH/gL, but not gH/gL/gO, mediate entry into fibroblasts [20], [21], while both gH/gL and gH/gL/pUL128-131 are necessary for virus entry into epithelial and endothelial cells [22].

Following natural infection, the first wave of the humoral response is characterized by antibodies able to neutralize the infection of endothelial/epithelial cells, in contrast to antibodies able to neutralize infection of fibroblasts [23]. By analyzing the antibody response to the pentameric complex in primary HCMV infections using cells transfected with multiple adenovirus vectors, each carrying a single gene of the pentamer, we found that the IgG antibody response to UL128-131 gene products is generally superior to the response to gH and appeared to follow the neutralizing antibody response [24]. These findings support the hypothesis that the HCMV pentamer glycoprotein complex may be an important target of neutralizing antibodies. In line with this hypothesis is the observation that the Towne vaccine (lacking a functional pentamer complex) and the gB/MF59 subunit vaccine, both characterized by partial efficacy, induce an epithelial entry-specific neutralizing activity that is on average 28-fold (Towne) or 15-fold (gB/MF59) lower than that observed after natural infection [25].

In the present study, we took advantage of new serological assays, developed using human monoclonal antibodies (mAbs) and soluble recombinant dimer and pentameric complexes [14], [26], to dissect the antibody response to HCMV glycoproteins in transmitter and non-transmitter pregnant women. The results demonstrate that in primary infections, the immune response to the pentamer gH/gL/pUL128-131 complex is predominant, and that the early presence of neutralizing antibodies directed to multiple sites on the pentamer is associated with a reduced risk of HCMV vertical transmission.

## Materials and Methods

### Ethics Statement

The study was approved by the Fondazione IRCCS Policlinico San Matteo Bioethics Committee, and informed written consent was obtained from each subject included in the study.

### Patients

The antibody response to the HCMV glycoprotein complex and its components was investigated in serum samples drawn from: i) a group of 13 HCMV-seronegative healthy blood donors; ii) a group of 20 HCMV-seropositive healthy blood donors; and iii) a group of 46 subjects (43 pregnant women and 3 non-pregnant subjects) with primary HCMV infection, in whom sequential serum samples were drawn at periodic medical visits at the Fondazione IRCCS Policlinico San Matteo, Pavia, Italy. Pregnant women with primary infection were identified among women referred to our Institution for suspected HCMV infection in pregnancy by using a battery of tests, some of which were commercially available and some developed in the laboratory. In detail, HCMV-specific IgG and IgM were determined by ETI-CYTOK-G and ETI-CYTOK-M (DiaSorin Saluggia, Italy), respectively. Presence of virus-specific IgM was confirmed by an in-house developed confirmatory ELA assay [27]. IgG avidity was determined by an in-house developed ELISA test [28]. In addition, neutralizing antibodies were routinely determined on human embryonic fibroblasts as well as ARPE-19 epithelial cells using the prototype HCMV strain VR 1814 [23]. HCMV in blood of pregnant women was searched for by real-time PCR (detection limit 25 copies/ml whole blood) and by pp65 antigenemia determination on cytospin preparations of 2×10^5^ peripheral blood leukocytes [29].

Primary HCMV infection was diagnosed based on the presence of at least two of the following four criteria: HCMV-specific IgG seroconversion, presence of virus-specificc IgM antibody, low IgG avidity index (AI), and DNAemia [27]–[29]. Recently, quantitative DNAemia results expressed as HCMV DNA copies/ml blood were translated into IU/ml with reference to the WHO International HCMV DNA Standard [30]. Timing of infection onset was achieved in the great majority of women based on the presence of a HCMV-specific seroconversion (within an interval of 1–2 months between the last seronegative and the first seropositive serum sample) and/or other serologic (IgM antibody and IgG AI) and virologic (DNAemia) findings, associated with presence of clinical signs/symptoms. In the minority of women, in whom signs/symptoms were not observed, IgG and IgM antibody kinetics were considered in association with low IgG AI for determination of infection onset timing [31].

HCMV transmission was diagnosed either antenatally by detection of viral DNA in and virus isolation from amniotic fluid samples or by virus isolation from urine samples collected within the first 2 weeks of life.

Of the 43 pregnant women, 19 transmitted and 22 did not transmit the infection to the fetus. Regarding the two remaining women, for one, transmission was unknown due to pregnancy termination, while the second had a delayed transmission to the fetus (amniotic fluid was negative but the newborn was infected at birth). Among the 43 pregnant women, 23 were selected (11 non-transmitters, and 12 transmitters) for the study of serum antibody titers against HCMV glycoprotein complexes and site-specific reactivity, based on the availability of multiple sequential serum samples, with the first available sample drawn within 60 days after onset of infection and available data sufficient to estimate the date of infection onset with a high probability. Timing of infection onset was achieved in 21/23 of these women based on the presence of clinical signs/symptoms associated to serologic data. In the remaining 2 women, in whom signs/symptoms were not observed, onset of infection was approximately assigned to a time point in the middle of the 1-month interval between the first positive and the last negative HCMV-specific IgG serum sample.

### Preparation and Purification of gH/gL/pUL128-131, gH/gL and gB

As previously reported, intronless, full-length UL128, UL130, UL131, gH, gL, gO, and gB were cloned into pcDNA3 vectors (Invitrogen) by PCR with *Pfu* turbo on cDNA of VR1814-infected MRC-9 cells, using primers introducing the desired restriction sites [14]. In order to obtain the secreted soluble forms of the glycoproteins, the transmembrane portion and the cytoplasmic domains were removed from gH and gB genes [16], [32]. Then, the gH/gL/pUL128-131 complex was obtained by co-transfecting the cells with UL128, UL130, UL131, gL, and gH plasmids with a mass ratio of 0.6∶0.6∶0.6∶0.8∶ 1, while the gH/gL complex was obtained by co-transfecting the cells with gH, gL and gO plasmids with a mass ratio of 1∶ 1.2∶0.8. For purification purposes 6x Histag was added to the C-terminus of UL131, gH, and gB, for gH/gL/pUL128-131, gH/gL and gB, respectively.

Constructs were used to transfect HEK293F cells (Invitrogen) with DNA and polyethyleneimine MAX (Polysciences) premixed in Opti-PRO SFM medium (Invitrogen). After 10 days culture the supernatant was harvested, and the presence of the proper HCMV glycoprotein complex was verified by ELISA using human mAbs specific for the different neutralization sites of the complex [14]. Glycoprotein complexes were purified on Histrap HP columns and subsequently on a Superdex 200 gel filtration column (GE Healthcare) according to the manufacturer’s instructions. Recombinant gB, gH/gL and gH/gL/UL128-131 had apparent molecular weights of 200–300 kDa. Fractions containing the protein of interest were merged and concentrated using ultrafiltration 30K columns (Sartorius Stedim Biotech, Goettingen, Germany). SDS-PAGE and Western blot analysis of recombinant proteins confirmed their identity and purity >80% (see Results).

### Determination of IgG Antibodies to the Pentamer, gH/gL and gB by ELISA

Half-area 96-well polystyrene plates (Corning) were coated overnight with an in-house developed murine anti-gH mAb (mH1P73), or an anti-gB mAb (HCMV37, Abcam, Cambridge, UK) and blocked with 5% skimmed milk in PBS, as recently reported [26]. After a double wash with PBS-0.05% Tween20, ELISA plates were incubated for 90 min with cell culture supernatants containing the pentamer, gH/gL complex or gB released from transfected cells. Following two washings, human serum (in 5% skimmed milk) was added at a single 1∶50 dilution or in serial two-fold dilutions and incubated 1 h at RT. After four washings, the horseradish peroxidase-labeled goat IgG fraction to human IgG (Fc-chain-specific) was added and incubated 45 min at room temperature, prior to adding the substrate solution. Net OD was obtained by subtracting the OD value obtained by incubating the serum without antigen from the value given by the serum incubated in the presence of antigen.

In order to equalize the amount of pentamer and gH/gL dimer bound to the solid phase and assuming that the different components of the pentamer were stoichiometrically represented at a ratio of 1∶1:1∶1∶1, the two preparations were tested in a capture ELISA system and their dilutions were normalized in order to obtain equal OD values when tested with an anti-gH primary mAb. Subsequently, 23 HCMV-seronegative and 10 HCMV-seropositive healthy blood donors were tested in a capture ELISA system to establish a cut-off indicating the reactivity of human sera with the pentamer or the dimer gH/gL. The mean value +2SD gave a cut-off of 0.10 (OD) for both the pentamer and gH/gL and a cut-off of 0.20 for gB antibodies.

### Inhibition of mAb Binding (IMAB) by Competitive Human Sera

The study of the reactivity of sequential human sera (from 11 non-transmitter and 12 transmitter pregnant women with primary HCMV infection) with previously identified neutralization sites of glycoproteins forming the pentamer complex was performed by using a competitive binding assay [26], [33]. In this assay, human sera containing antibodies to a defined neutralization site prevented binding of the relevant murinized site-specific mAb. Briefly, following coating of a human mAb (anti-gH, 3G16) to the solid-phase, and blocking of non-specific binding sites with 5% skimmed milk in PBS, the complex was captured after a 90 min incubation at RT. Human serum (or mAb) was added in serial two-fold dilutions in duplicate (starting from 1∶5) and incubated 1 h at RT, prior to adding a primary murinized mAb (at a concentration corresponding to 80% of its maximal OD reactivity) for 1 h at RT, and then a secondary alkaline phosphatase-labeled goat anti-mouse IgG (γ-chain-specific) for 45 min at RT and, finally, the substrate solution. The % inhibition was calculated as follows: (OD w/o serum-OD w serum)/(OD w/o serum-OD background)×100. To calculate the IMAB_50_ titer, a dose-response curve plotting log_10_ serum dilution *vs* % inhibition was constructed, from which the serum dilution corresponding to the IMAB_50_ titer was interpolated. By using this curve, it was possible to interpolate positivities between 1∶1 (undiluted serum) and 1∶5 (first tested serum dilution).

### Neutralization Assay

Serial dilutions of heat-inactivated human sera were incubated in duplicate for 60 min at 37°C with an equal virus volume containing 100 infectious units of VR1814 [23]. Virus-antibody mixtures were then added in duplicate to monolayers of ARPE-19 or HELF cells, and centrifuged at 700×*g* for 30 min. After 48 h incubation, cells were fixed and stained for HCMV p72 using a pool of murine mAbs [34]. The serum dilution inhibiting virus infectivity by 50% or more with respect to virus controls was reported as the neutralizing-antibody titer.

### Plaque Formation Inhibition (PFI) and Leukocyte Transfer Inhibition (LTI) by Human mAbs and Sera

Using serial dilutions of human mAbs or human sera from patients with primary HCMV infection, PFI in ARPE-19 cells as well as LTI from HCMV-infected HUVEC was investigated, as previously reported [23], [26].

PFI was investigated as follows. Following virus absorption (50 pfu) by centrifugation for 30 min at 700×*g* at RT, 96-well microplate cell cultures were washed and supplemented with medium containing serial dilutions of mAbs or serum for 120 h. Cells were then fixed and stained by using a p72-specific mAb pool [34]. The percent PFI was determined by dividing the difference between the number of viral plaques in the absence or presence of mAb (or human serum) by the number of plaques in the absence of antibody (×100). All experiments were done in triplicate.

LTI experiments were performed in 24-well microplates by incubating human mAbs for 2 h at 37°C with monolayers of VR1814-infected HUVEC (96 h pi) prior to and overnight during co-cultivation with leukocytes [23], [35]. After co-culture, leukocytes were purified by migration through a Transwell device (Costar). Control experiments were done in the absence of serum or mAbs. Leukocytes were then fixed, permeabilized and stained with a pool of pp65-specific murine mAbs, as reported [36]. The percent inhibition was determined by dividing the difference between the number of pp65-positive leukocytes in co-culture experiments done in the absence or presence of antibody by the number in the absence of antibody (×100).

To calculate PFI_50_ and LTI_50_ titers, a dose-response curve plotting % inhibition *vs* mAb concentration (or serum dilution) was constructed, from which the antibody dilution corresponding to PFI_50_ or LTI_50_ titer was interpolated.

### Statistical Analysis

Non-linear regression models were used to express the kinetics of different serological parameters (IgG antibodies to gH/gL/pUL128-131, gH/gL and gB; neutralizing antibodies and PFI_50_ titers). The curves were compared by the extra-sum-of square F test (GraphPad Prism 5.0 software). The relative time to appearance of sera reactivity to the different neutralization sites was analyzed by the Kaplan-Meyer method and compared by the log-rank test. IMAB_50_ titers to the different neutralization sites, numbers of pentamer epitopes recognized and HCMV DNA load were compared by the Mann-Whitney U-test, whereas the proportion of patients with HCMV DNA in blood was compared by the Fisher’s exact test. Linear regression was used to calculate the correlation between log_10_ neutralizing and ELISA IgG antibody titers.

## Results

### Production and Characterization of Soluble HCMV Glycoprotein Complexes

**Figure 1:**
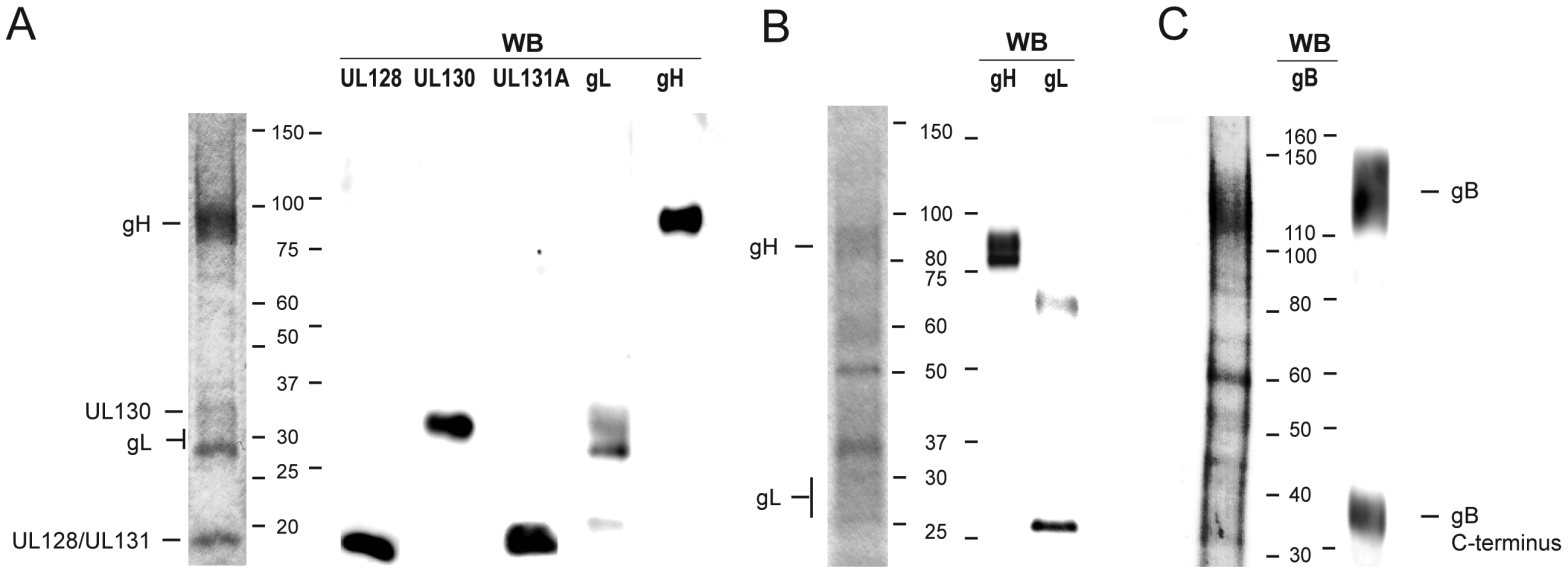
SDS-PAGE and Western blot (WB) analysis of soluble HCMV glycoprotein complexes. (A) Pentameric gH/gL/pUL128-131 complex, (B) gH/gL and (C) gB complexes were subjected to SDS-PAGE and Western blotting. Samples were stained with Coomassie blue (left panels) or probed with specific antibodies after WB (right panels). (B) A band of about 60 kDa on gL blot is apparently a co-purified contaminant recognized by anti-gL polyclonal serum raised in rabbits. (C) Soluble gB exists in full-length and truncated forms that associate in dimeric complex, as described in Carlson et al. (32). N-terminus of truncated gB form is not detected since gB was his-tagged on the C-terminus and the blot was probed with anti-histag antibody.

To characterize patient response to HCMV glycoprotein complexes, we produced recombinant soluble pentamer, dimer and gB by transiently transfecting 293F HEK cells and purifying his-tagged proteins from cell culture supernatants. SDS-PAGE and Western blot analysis with specific antibodies confirmed the identity of proteins recovered (Fig. 1).

### Early Production of Pentamer-specific Antibodies that Neutralize Epithelial Cell Infection

**Figure 2:**
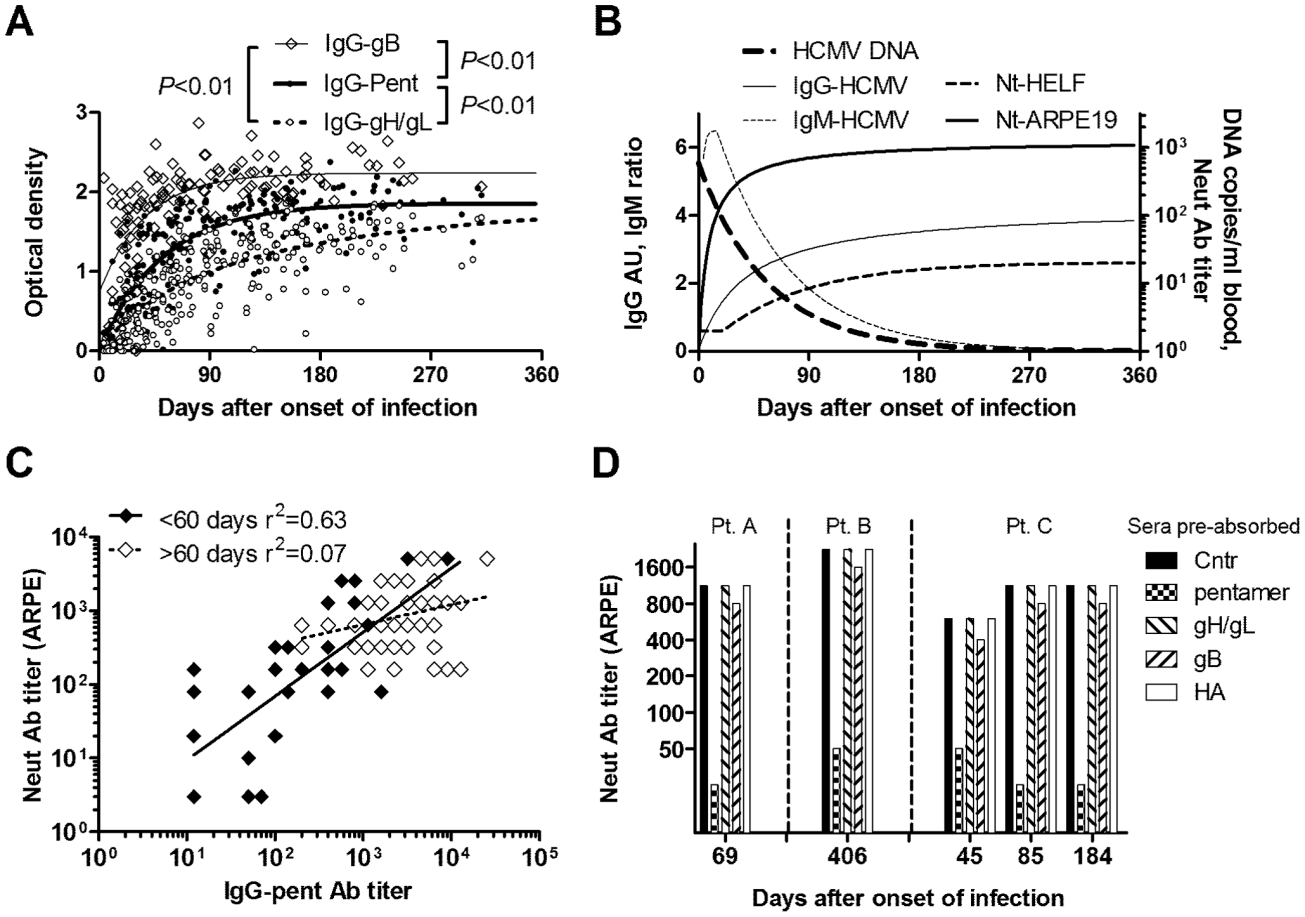
Kinetics of the antibody response to primary HCMV infection. (A) Kinetics of the appearance of anti-pentamer (IgG-Pent), anti-gH/gL (IgG-gH/gL) and anti-gB (IgG-gB) antibodies in 46 subjects with primary HCMV infection. Shown are individual values and non-linear regression curves for the three specificities. *P*-values were calculated using the extra-sum-of square F test. (B) Kinetics of the mean HCMV serological response in the 46 patients’ sera. IgG-HCMV refers to HCMV lysate-specific IgG Arbitrary Units (AU); IgM-HCMV refers to HCMV lysate-specific IgM ratio; Nt-HELF and Nt-ARPE19 refer to neutralizing antibody (Neut Ab) titer on human embryonic lung fibroblasts (HELF) and epithelial (ARPE-19) cells, respectively; HCMV DNA refers to viral load in blood. (C) Correlation between IgG-pentamer antibody titers measured by ELISA and neutralizing antibody titers measured using ARPE-19. Values were obtained from sera collected <60 day post infection (filled diamonds) or >60 days post infection (empty diamonds). The regression coefficient (r^2^) is also shown. (D) Neutralizing antibody (Neut Ab) titers of sera from 3 patients (Pt.A, Pt.B, and Pt.C) were measured using ARPE-19. Sera were used either untreated or after absorption with the soluble pentameric complex, gH/gL dimer, or gB proteins from HCMV or with influenza A virus hemagglutinin (HA).

The kinetics of the antibody response to different HCMV antigens and the serum capacity to neutralize infection of fibroblasts or epithelial cells was investigated in 43 pregnant women and 3 non-pregnant women during the first year after onset of primary HCMV infection. Soluble forms of the gH/gL/UL128-131 pentamer complex, gH/gL dimer and gB were produced in transfected cells, and used to measure specific IgG by ELISA in longitudinal serum samples (median number 5, range 3 to 9 sera/subject, n = 240 sera). As shown in Figure 2A, antibodies to gB increased rapidly and to significantly higher levels as compared to those against the pentamer, while antibodies to gH/gL showed significantly slower kinetics. Serum antibodies capable of neutralizing infection of an epithelial cell line (ARPE-19) appeared early and rapidly reached high titers. In contrast, serum antibodies capable of neutralizing infection of fibroblasts (HELF) appeared later and peaked only at approximately 6 months after onset of infection, showing titers lower by 2–3 log_10_ (Fig. 2B). As total IgG antibodies to HCMV lysate increased, HCMV DNA as well as IgM antibodies to HCMV lysate declined in the first 3–6 months after infection (Fig. 2B).

We then investigated the existence of a correlation between anti-pentamer ELISA-IgG antibodies and titers of serum antibodies able to neutralize epithelial cell infection. As shown in Fig. 2C, there was a good correlation between anti-pentamer antibody levels and neutralizing titers in the first 2 months after onset of infection, which was lost at later time points (>60 days) after onset of infection.

To investigate the relative contribution of antibodies with different specificities to viral neutralization, we absorbed pentamer-, gH/gL- or gB-specific antibodies from the sera of 3 patients by adding the relevant purified antigens or influenza hemagglutin (HA) as a control. Depletion was specific and efficient (greater than 90%) as shown by specific ELISA (Table 1). Strikingly, sera pre-adsorbed with the pentameric complex showed a highly reduced capacity (>90%) to neutralize viral infection of ARPE-19, even when collected at late time points (184 or 406 days after onset of infection), while their activity was substantially unaffected when pre-adsorbed with gH/gL or gB (Fig. 2D). Taken together, these data suggest that the neutralizing activity in sera of pregnant women with primary HCMV infection is conferred by antibodies against the pentameric gH/gL/pUL128-131 complex.

**Table 1:** Effect of serum absorption with purified HCMV proteins on ELISA IgG antibody titer.

ELISA IgG Ab titer to	ELISA titer (% depletion) following absorption with purified			
	pentamer	gH/gL	gB	HA^a^
pentamer (1∶3,200)	1∶50 (99)	1∶800 (75)	1∶3,200 (0)	1∶3,200 (0)
gH/gL (1∶1,600)	1∶100 (94)	1∶100 (94)	1∶1,600 (0)	1∶1,600 (0)
gB (1∶12,800)	1∶12,800 (0)	1∶12,800 (0)	1∶800 (94)	1∶12,800 (0)

a Influenza A hemagglutinin.

### Kinetics of Serum Antibodies in Transmitter and Non-transmitter Women

**Figure 3:**
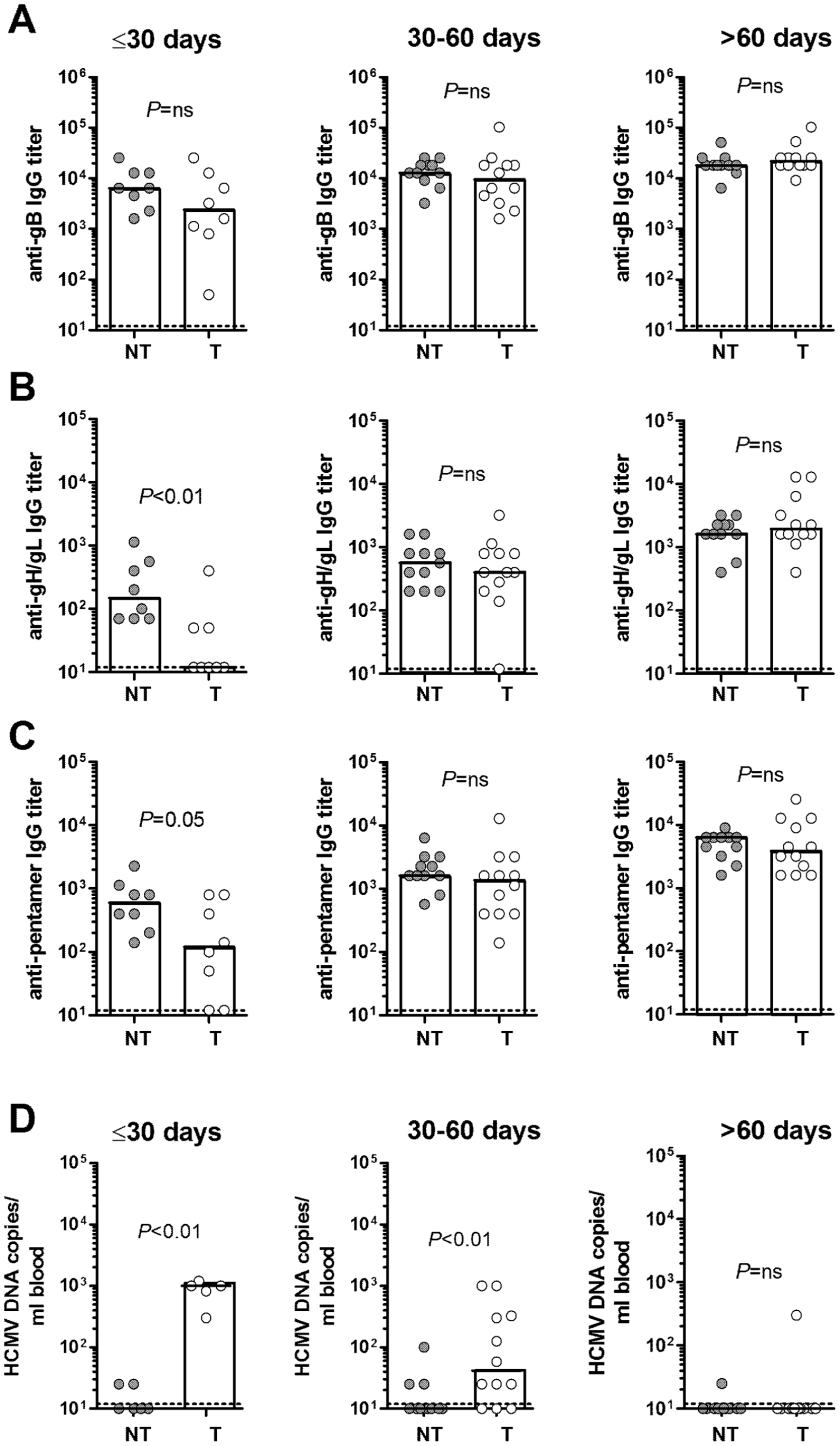
IgG antibody titers and DNA viral load at three time points of primary HCMV infection. IgG antibody titers in 11 non-transmitter (NT) and 12 transmitter (T) mothers against gB (A), gH/gL (B), or pentamer (C) at three different time intervals (≤30 days, 30–60 days, >60 days) after onset of infection. (D) HCMV DNA copies/ml blood in the same sera samples at the three time intervals. Dotted lines represent the detection limit of the assays. *P*-values were calculated using the Mann-Whitney U-test (A-C) or the Fisher’s exact test (D).

We next compared the kinetics of serum antibodies in 11 women who did not transmit the virus to the fetus (non-transmitter) and 12 women who transmitted the virus (transmitter), as reported in Table 2. In both groups of patients, IgG antibody titers against gB increased rapidly with overlapping kinetics, reaching a plateau within the first 30 days after infection (Fig. S1A). In contrast, IgG antibodies to the gH/gL dimer and the pentamer appeared earlier and reached higher titers in non-transmitter women compared to transmitter women (Fig. S1B, C). Accordingly, no significant differences in gB antibody titers in transmitters and non-transmitters were observed when the data were examined by grouping them in three time intervals (Fig. 3A), while IgG antibody titers against the pentamer and the gH/gL dimer were significantly higher in non-transmitter women compared to transmitter women in the first 30 days after onset of infection although, at later time points (31–60 days and >60 days), titers increased in both groups reaching comparable levels (Fig. 3B, C). Interestingly, the number of HCMV DNA copies/ml blood was significantly higher in transmitters as compared to non-transmitters, both in the first (median 1,000 DNA copies/ml blood *vs* undetectable) and second (median 42 DNA copies/ml blood *vs* undetectable) time interval (≤30 days and 31–60 days after onset), whereas no difference was observed between the two groups in the >60 day interval (Fig. 3D).

**Table 2:** Characteristics of the 23 pregnant women analyzed for antibodies against neutralization sites in the pentameric complex.

Parameter	Non-transmitter mothers (n = 11)	Transmitter mothers (n = 12)	*P*
Gestational week at onset of infection, median (range)	10 (6–16)	7 (4–26)	ns^a^
No. symptomatic infection (%)	9 (82)	12 (100)	ns^a^
No. samples available, median (range)	5 (3–9)	6 (4–8)	ns^a^
Serologic parameters utilized for dating infection onset, no. cases (%)			
-Seroconversion	5 (45)	7 (58)	ns^b^
-IgM kinetics	5 (45)	4 (33)	ns^b^
-IgG avidity kinetics	4 (36)	2 (17)	ns^b^
Median time to HCMV DNAemia clearance from blood (days)	32	93	<0.01^c^
No. pregnancy terminations (%)	1 (9)^d^	1 (8)	ns^b^

a Mann-Whitney U test.

b Fisher’s exact test.

c Log-rank test.

d Termination of pregnancy due to severe fetal malformations not related to HCMV infection.

Taken together, these results indicate that non-transmitter mothers are characterized by a more rapid IgG antibody response to the pentameric complex and the gH/gL dimer and that this early antibody response correlates with more rapid control of viral infection.

### Distinct Kinetics of Antibody Responses to 10 Neutralization Sites of the Pentamer in Non-transmitter and Transmitter Women

We have previously isolated from human memory B cells a panel of neutralizing monoclonal antibodies that target distinct sites on the pentameric complex [14]. To analyze the fine-specificity of the serum anti-pentamer antibodies in transmitter and non-transmitter mothers, we measured the capacity of the sera to inhibit binding of the monoclonal antibody panel to plate-bound pentamer (Fig. S2). Using the IMAB assay, we were able to define the serum titers of antibodies to 10 different sites: site 1 (defined by mAb 15D8 that binds to pUL128); sites 2, 3 and 4 (defined by human mAbs 1F11, 4N10, and 10P3 that bind to three non-overlapping epitopes of the dimer pUL130-131); sites 5 and 6 (defined by human mAbs 6G4, and 7I13 that bind to the trimer pUL128-130-131); site 7 (defined by human mAb 8I21 that recognizes an epitope shared by gH/gL/UL128-130); sites 8, 9, and 10 (defined by human mAbs 3G16, 11B12, and H1P73 that recognize non-overlapping epitopes on gH). The specificity of the assay was demonstrated by lack of IMAB reactivity of an irrelevant mAb (specific for HA) and by the fact that none of the HCMV-seronegative blood donors tested showed reactivity with any of the neutralization sites studied, while all of the HCMV-seropositive blood donors with remote HCMV infection showed reactivity with each of the 10 neutralization sites tested.

**Figure 4:**
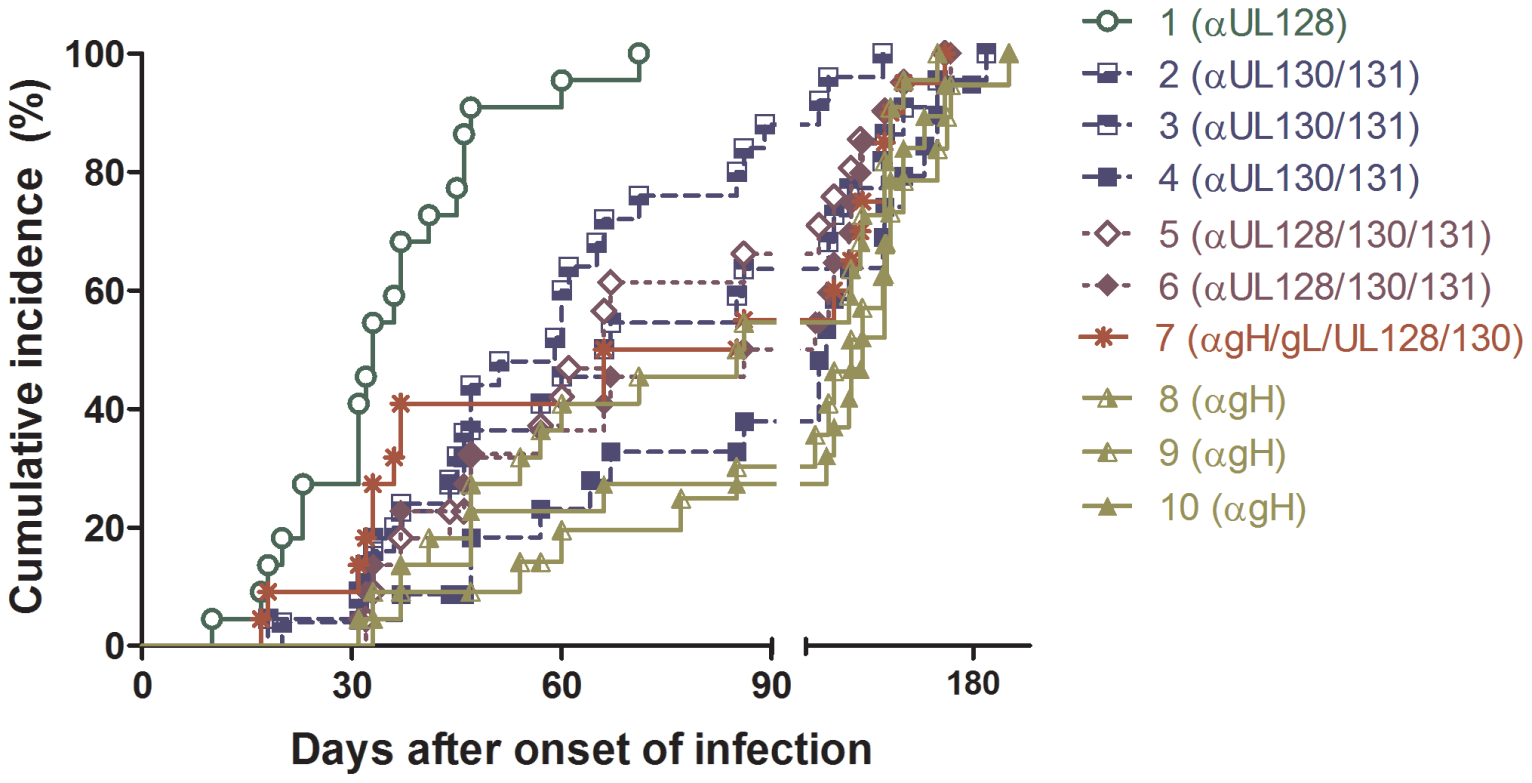
Cumulative antibody response to the pentamer neutralization sites. Patients’ antibody response to neutralizing sites of the pentamer estimated *via* inhibition of monoclonal antibody binding (IMAB) assay using a panel of 10 human monoclonal antibodies with defined specificity for different sites on the HCMV pentamer complex, as indicated. The cumulative incidence of site-specific antibodies in 23 pregnant women with primary HCMV infection during the entire follow-up period is shown.

The IMAB assay was performed on serum samples from 23 pregnant women (11 non-transmitters, and 12 transmitters). When the cumulative kinetics of the antibody response to all neutralization sites was examined, it was found that the antibody response to pUL128 (site 1) appeared significantly earlier (P<0.01) than those to the other pentamer antigenic sites, whereas antibodies to site 4 (anti-pUL130-131), and sites 9 and 10 (anti-gH) were detected significantly later (P<0.05) as compared to antibodies to sites 2, 3, and 5 to 8 (Fig. 4). These findings highlight an individual difference in the production of antibodies to the different sites of the pentameric complex.

**Figure 5:**
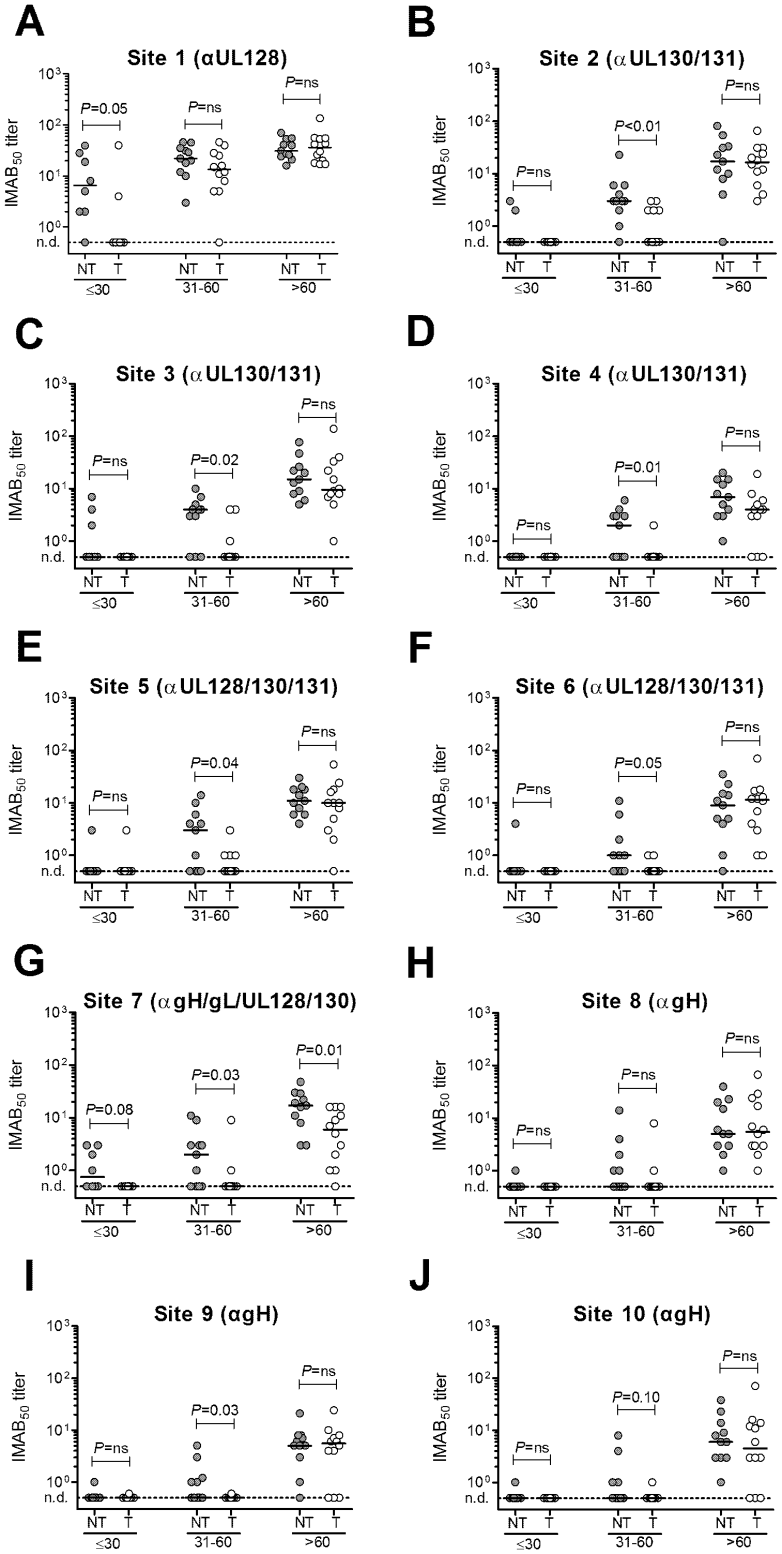
IMAB_50_ titers by site-specific antibodies present in human sera from primary HCMV infection. IMAB_50_ titers of neutralizing site-specific IgG antibodies in 12 transmitter (T) and 11 non-transmitter (NT) mothers at three different time intervals (≤30 days, 30–60 days, >60 days) after onset of infection. (A-J): Sites 1 to 10 as defined by the indicated human monoclonal antibodies to gH/gL/pUL128-130-131 [14]. Dotted lines represent the detection limit of the assays. *P*-values were calculated using the Mann-Whitney U-test; n.d., not detected.

We next compared the IMAB_50_ titers for the 10 antigenic sites in the two groups of transmitter and non-transmitter women examined according to the three time intervals (≤30, 31–60, >60 days after onset, Fig. 5). Serum samples were available for 8 transmitter and 8 non-transmitter women in the first time interval, and for 12 transmitter and 11 non-transmitter women at the other time intervals analyzed. In blood samples collected during the second month after the onset of infection (median sampling time 47, range 31–60, days for NT women *vs* 46, range 31–60, days for T women) the IMAB_50_ titer was significantly lower in the group of transmitters as compared to the group of non-transmitters for most of the sites investigated (except for sites 1, 8 and 10). In addition, IMAB_50_ titers to site 1 were significantly higher in non-transmitters in the ≤30 days time interval (Fig. 5A), and antibodies to site 7 (Fig. 5G) remained significantly higher in this group also at the late time point examined (>60 days), when reactivity to the other sites was no longer different between the two groups. In order to exclude a possible bias due to different criteria used for timing, we repeated the analysis after exclusion of the two patients in whom the onset infection was dated on the basis of serologic data only (IgG seroconversion) in the absence of symptoms, in order to exclude a possible bias due to different criteria used for timing. Except for site 6, the IMAB_50_ titers to the other sites remained significantly lower in sera from T women (data not shown).

### The Breadth of Anti-pentamer Antibodies is Associated with Reduced Rate of Intrauterine Transmission

**Figure 6:**
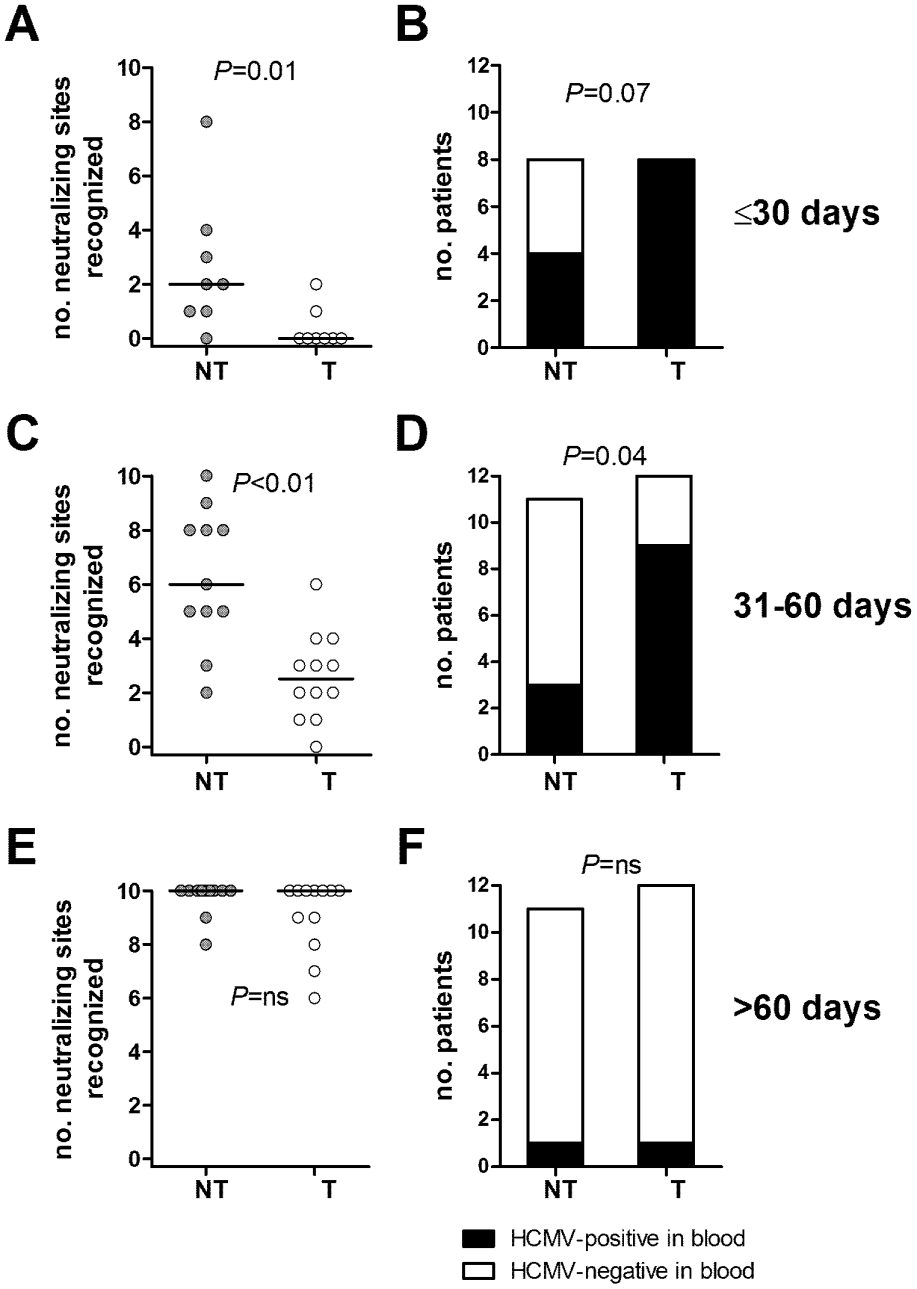
Overall number of neutralization sites of the pentamer reactive with human sera and viral load at three time points. Number of neutralization sites recognized by sera from 11 non-transmitter (NT) and 12 transmitter (T) mothers at (A) less than 30 days, (C) 31–60 days, and (E) more than 60 days after onset of infection. (B, D, F): Number of HCMV-DNA positive (black bars) and HCMV-DNA negative (white bars) women in the NT and T groups at the same time intervals. *P*-values were calculated using the Mann-Whitney U-test (A, C, E) or the Fisher’s exact test (B, D, F).

We next compared the breadth of the anti-pentamer response in non-transmitter and transmitter women and whether there was an association with control of viral infection. As shown in Fig. 6A, C, the number of neutralization sites recognized by transmitter women was significantly lower than the number of sites recognized by non-transmitter women during both the first and second month after onset of infection, while no differences were found in the >60 day interval (Fig. 6E). Furthermore, the number of women positive for HCMV DNA in blood was higher among transmitter women as compared to non-transmitter women, both in the first (positive women 8/8 among transmitter *vs* 4/8 among non-transmitter women, P = 0.07) and second (positive women 9/12 among transmitter *vs* 3/11 among non-transmitter women, P = 0.04) month after onset (Fig. 6B, D). No difference (positive women 1/12 among transmitter *vs* 1/11 among non-transmitter women, P = ns) was observed between the two groups in the >60 day interval (Fig. 6F).

### Blocking of Viral Spread and Transfer to Leukocytes by Neutralizing Human mAbs and Human Sera

Lastly, we tested mAbs of defined specificity and serum samples from transmitter and non-transmitter women for their capacity to inhibit cell-to-cell spread (PFI_50_) of HCMV in ARPE-19 cell monolayers.

**Figure 7:**
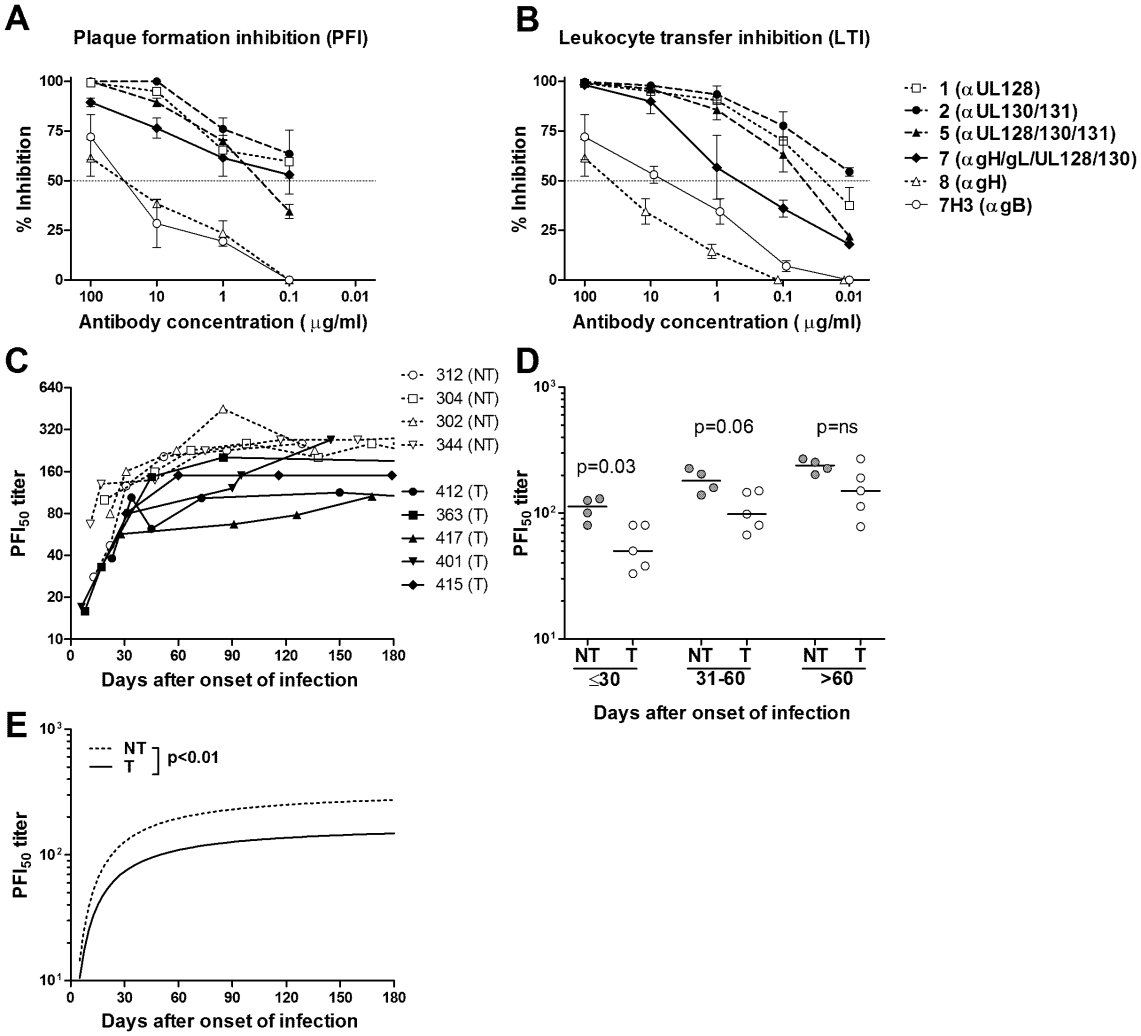
Plaque formation and leukocyte transfer inhibition by human monoclonal and serum antibodies. Dose-effect inhibition of (A) HCMV plaque formation (PFI) in ARPE-19 cells, and (B) HCMV transfer to leukocytes (LTI) from infected HUVEC cells by serial concentrations of human mAbs specific for different sites on the pentamer complex. An anti-gB mAb (7H3) was used as a control. (A) mAbs to gB and gH show a PFI_50_ activity at a concentration about a thousand-fold higher in comparison to human mAbs directed to pUL128-131. (B) Comparable LTI_50_ activity was displayed by human mAbs directed to pUL128-131, whereas mAbs to gH and gB again show a much weaker inhibitory effect. (C) Individual PFI_50_ titers in sequential sera collected within the first 3 months after onset of infection in 5 transmitter (T) and 4 non-transmitter (NT) pregnant women. (D) PFI_50_ titers at three different time intervals (≤30 days, 30–60 days, >60 days) after onset of infection are shown. *P*-values were calculated using the Mann-Whitney U-test. (E) Regression curves of PFI_50_ titers in transmitter (T) and non-transmitter (NT) women. *P*-value was calculated using the extra-sum-of square F test.

Consistently, mAbs directed against the pentamer showed greater inhibition in comparison with antibodies directed against gH or gB (Fig. 7A). Conversely, only antibodies against gB or gH blocked virus spread in HELF cells, in the absence of any significant blocking effect by antibodies directed to the pentamer or UL128-131 gene products (data not shown). We also investigated *in vitro* the inhibitory effect of human mAbs on the transfer of virus from HUVEC to leukocytes (LTI_50_) [23]. We found that, within the concentration range of 100 to 0.1 µg/ml, most neutralizing antibodies displayed significant blocking of virus transfer to leukocytes in the range of 100% (100 µg/ml) to 70% (0.1 µg/ml). This blocking effect was characteristic of antibodies that bind exclusively to the pentamer, but not of antibodies that bind to gH/gL or gB (Fig. 7B). A similar PFI effect was observed in sequential sera from 5 transmitter and 4 non-transmitter women with primary HCMV infection (Fig. 7C). The PFI_50_ was significantly higher in non-transmitter women during the first month after onset and close to significance (P = 0.06) in the second month after onset (Fig. 7D). Similarly, the average kinetics of the PFI_50_ titer in non-transmitter women increased significantly faster and reached a higher level as compared to transmitter women (Fig. 7E).

Taken together, these findings indicate that the neutralizing activity of both human mAbs and human sera is able to inhibit both cell-to-cell spread and virus transfer to leukocytes, thus inhibiting virus dissemination by two different mechanisms.

## Discussion

In this study, we report that early detection of HCMV neutralizing antibodies directed against the pentameric complex gH/gL/pUL128-131 and, in particular, to the UL128-131 gene products appears to be associated with a lower rate of HCMV transmission from the mother to the fetus. This was shown by the finding that non-transmitter mothers exhibit an earlier antibody response to different antigenic sites of the pentamer as compared to transmitter mothers. The biological relevance of anti-pentamer antibodies is in parallel highlighted by the finding that antibodies to gB are produced by non-transmitter and transmitter mothers with the same kinetics and in comparable amounts. This latter aspect also excludes any systematic error in determining the time of infection that might have biased our sampling.

The important role of antibodies directed against UL128-131 locus gene products is also supported by the following findings: i) the absorption of sera with recombinant soluble pentamer strongly reduced serum neutralizing activity, while absorption with gH/gL or gB had minimal effects, consistent with data obtained using hyper-immune globulin preparations [37]; ii) mAbs targeting the pUL128-131 components of the pentamer showed a thousand-fold greater neutralizing activity as compared to mAbs specific for gH or gB [14]; iii) anti-pentamer antibodies potently inhibit infection of epithelial, endothelial and myeloid cells, thus interfering with viral spread *in vivo* and potential virus transmission to the placenta and the fetus, and iv) during the first two months after onset of infection there was a significant correlation between the titers of serum IgG antibodies to the pentamer and neutralizing titers determined in epithelial cells. The reduced correlation observed in the subsequent period may be due to the appearance of pentamer-specific antibodies without neutralizing activity, as well as to the appearance of neutralizing antibodies with different specificity. On the other hand, the lack of correlation between gB-specific antibodies and control of HCMV transmission to the fetus may be due to the fact that the great majority of antibodies elicited by gB do not neutralize HCMV infection [38], whereas we have evidence that most human pentamer-specific antibodies are neutralizing (A Kabanova, unpublished data).

Conventional ELISA measurement of sera reactivity with crude viral antigens does not discriminate between antibodies playing a primary role in antiviral immunity (i.e. those recognizing neutralization sites) and antibodies reactive with non-functional sites. In this study, we used the IMAB assay that allowed us to study the kinetics of antibodies directed against 10 neutralization sites on the pentameric complex that were previously identified by a panel of human mAbs [14]. This site-specific analysis revealed that in non-transmitter mothers antibodies targeting 8 out of 10 sites appeared with faster kinetics compared to transmitter mothers. Thus, the IMAB assay appears efficacious in revealing differential kinetics of site-specific neutralizing antibodies in the two groups of mothers in the early phase after onset of infection, thus representing a useful diagnostic parameter.

Neutralizing antibodies are thought to act primarily by preventing cell-free virus from infecting susceptible cells. However, the main route of HCMV dissemination *in vivo* is associated with viral spread by infected polymorphonuclear leukocytes and monocytes [39]. Here, we have shown that mAbs to the pentamer sites as well as sera containing such antibodies effectively inhibited viral spread as measured by plaque formation [23]. Importantly, the capacity of serum to inhibit viral spread was significantly lower in transmitter as compared to non-transmitter women. An additional correlate of potential *in vivo* protection was studied by investigating the LTI_50_ activity (i.e. blocking HCMV transfer from endothelial cells to leukocytes) of human mAbs. In this case, all mAbs directed to the pentamer complex were able to block transfer from HUVEC to leukocytes. Thus, the pUL128-131 gene products appear to be involved in the mechanism of virus transfer to leukocytes and virus dissemination [39].

The delayed development in transmitter women of HCMV-specific T-cells with proliferative potential and the T_EMRA_ phenotype have been identified as two other risk factors predictive of virus transmission from mothers to fetus during pregnancy (7–10). During primary HCMV infection, the appearance in peripheral blood of T cells able to proliferate *in vitro* in response to a recall HCMV stimulus, as well as the development of T_EMRA_ cells were found to correlate with clearance of virus from blood [8], [9], [40]. Thus, it can be hypothesized that early development of antibodies to the neutralization sites of the pentamer complex, along with functional T cell response, synergistically induce a more rapid block of HCMV dissemination resulting in faster clearance of virus from blood. Alternatively, it could be hypothesized that a lower viral load after infection may determine both lack of transmission to the fetus and a faster immune response in the mother. However, it is more likely that the maternal immune response is responsible for the control of viral replication and dissemination and not *vice versa*.

Administration of human hyperimmune globulin (Ig) in pregnant women with primary HCMV infection has been claimed as useful for both prevention and therapy of congenital HCMV infection [11]. This treatment has also been reported to ameliorate placenta oxygenation and nutrition [41] and to be beneficial in decreasing the severity of disabilities caused by fetal HCMV infection after primary maternal infection during pregnancy [42]. In the present study, the major differences in antibodies reactive with the neutralization sites of the pentamer were detected between 30 and 60 days after onset of infection, whereas from 60 days onward the immunological parameters investigated were comparable between transmitter and non-transmitter women. These findings suggest that in pregnant women passive immunization (either by human Ig or human mAbs) might have an effect in the prevention of congenital HCMV infection, when administered in an early phase after infection or prophylactically in seronegative pregnant women.

In conclusion, the finding that an earlier antibody response to the neutralizing sites of the pentamer in non-transmitter mothers was associated with a reduced rate of HCMV vertical transmission indicates that human anti-pentamer neutralizing mAbs could be used for passive immunization of pregnant HCMV-seronegative women and that the HCMV pentamer complex is a favourable candidate for the development of an effective vaccine against HCMV.

## Supporting Information

Figure S1 **Kinetics of IgG antibodies to glycoprotein complexes.** (A-C). Kinetics of appearance of anti-gB (A), anti-gH/gL (B) and anti-pentamer (C) IgG antibodies in 11 transmitter (T, black symbols) and 12 non-transmitter (NT, white symbols) mothers. Individual values and non-linear regression curves in the two groups of patients are shown. *P*-value was calculated using the extra-sum-of square F test.
(TIF)

Figure S2 **Determination of IMAB_50_ titer on sequential sera from primary HCMV infection.** Inhibition of a human mAb anti-pUL128-131 (10P3, site 4-specific) binding by three sequential human sera collected from a pregnant woman with primary HCMV infection at different time points post-infection.
(TIF)
